# Enhancement of Photo-Oxidation Activities Depending on Structural Distortion of Fe-Doped TiO_2_ Nanoparticles

**DOI:** 10.1186/s11671-016-1263-6

**Published:** 2016-01-29

**Authors:** Yeonwoo Kim, Sena Yang, Eun Hee Jeon, Jaeyoon Baik, Namdong Kim, Hyun Sung Kim, Hangil Lee

**Affiliations:** Molecular-Level Interfaces Research Center, Department of Chemistry, KAIST, Daejeon, 34141 Republic of Korea; Beamline Research Division, Pohang Accelerator Laboratory (PAL), Pohang, 37673 Kyungbuk Republic of Korea; Department of Chemistry, Pukyoung National University, Busan, 48513 Republic of Korea; Department of Chemistry, Sookmyung Women’s University, Seoul, 04310 Republic of Korea

**Keywords:** Distorted TiO_2_, 2-Aminothiophenol, Photo-oxidation, Bandgap narrowing

## Abstract

To design a high-performance photocatalytic system with TiO_2_, it is necessary to reduce the bandgap and enhance the absorption efficiency. The reduction of the bandgap to the visible range was investigated with reference to the surface distortion of anatase TiO_2_ nanoparticles induced by varying Fe doping concentrations. Fe-doped TiO_2_ nanoparticles (Fe@TiO_2_) were synthesized by a hydrothermal method and analyzed by various surface analysis techniques such as transmission electron microscopy, Raman spectroscopy, X-ray diffraction, scanning transmission X-ray microscopy, and high-resolution photoemission spectroscopy. We observed that Fe doping over 5 wt.% gave rise to a distorted structure, i.e., Fe_2_Ti_3_O_9_, indicating numerous Ti^3+^ and oxygen-vacancy sites. The Ti^3+^ sites act as electron trap sites to deliver the electron to O_2_ as well as introduce the dopant level inside the bandgap, resulting in a significant increase in the photocatalytic oxidation reaction of thiol (–SH) of 2-aminothiophenol to sulfonic acid (–SO_3_H) under ultraviolet and visible light illumination.

**Electronic supplementary material:**

The online version of this article (doi:10.1186/s11671-016-1263-6) contains supplementary material, which is available to authorized users.

## Background

Titanium oxide (TiO_2_) is one of the most promising materials for various applications such as solar cells, gas sensors, photocatalysis, and corrosion protection due to its chemical stability, nontoxicity, and low cost [1, 2]. Furthermore, its high oxidation properties have been applied to the degradation of organic pollutants [3–5]. However, the problems caused by the large bandgap (3.0–3.2 eV) result in poor efficiency and limited light absorption in the visible region, which makes a practical application difficult [6, 7]. To solve these chronic problems and enhance catalytic properties, narrowing the bandgap is necessary.

One strategy is co-deposition of a noble metal such as Pt, Ag, Au, or Pd onto the TiO_2_ surface. This strategy has little effect on narrowing the bandgap, although it does improve the separation of holes and electrons significantly [8–13]. However, it is not cost effective and makes almost no contribution to light absorption in the visible range. Another strategy is the introduction of foreign atoms as dopants inside the TiO_2_ substrate [14–16]. This can narrow the TiO_2_ bandgap and change the electronic band structure, which can increase the photocatalytic performance significantly. If the effect is similar or the same as that of noble metals, transition metals such as Fe, Co, and Ni are the most feasible and cost-effective dopant candidates. Such transition metals possess various oxidation states and thus contribute to bandgap control.

We introduced Fe ions into the TiO_2_ substrate using a hydrothermal method and systematically investigated the photocatalytic activities of Fe-doped TiO_2_ nanoparticle (Fe@TiO_2_) at various Fe doping concentrations. Structural and electronic changes were characterized by surface analysis techniques. The photocatalytic activities were characterized by the oxidation of 2-aminothiophenol (2-ATP) with different light sources in the ultraviolet (365 nm) and visible (540 nm) regions. We found that 5 wt.% of Fe dopants in TiO_2_ nanoparticles form a new distorted phase in which catalytic performance is significantly enhanced by bandgap narrowing.

## Methods

### Materials

Titanium isopropoxide (TTIP, 99.9 %), tetramethyl ammonium hydroxide solution (TMAOH, 25 wt.% in H_2_O), and Fe(NO_3_)_3_ 9H_2_O (99.9 %) were purchased from Sigma-Aldrich and used as received.

### Preparation of Fe-Doped TiO_2_ Nanoparticles

#### Preparation of Dispersed Fe@TiO_2_ Nanoparticle Solution

TMAOH (1.2 g) was introduced into a round-bottomed flask (100 ml) containing double-distilled water (22.25 g). The diluted TMAOH solution was stirred for 10 min. TTIP (3.52 g, 12.4 mmol) was separately diluted with isopropanol (3.5 g) and stirred for 10 min. The diluted TTIP was added dropwise into the TMAOH solution with vigorous stirring at room temperature. Initially, white TiO_2_ precipitants appeared. Then, a desired amount of Fe(NO_3_)_3_ 9H_2_O (99.9 %) as a dopant was introduced into the synthetic gel solution. The round-bottomed flask containing the synthetic gel solution was placed in an oil bath. The temperature of the oil bath was maintained at 80 °C under continuous stirring. After approximately 10 min, the synthetic gel solution became a transparent solution. The synthetic gel was transferred to a Teflon-lined autoclave. The autoclave was placed in a convection oven preheated to 220 °C for 7 h. The produced Fe@TiO_2_ nanoparticles were collected by centrifugation at 10,000 rpm and washed with copious amounts of DDW to remove unreacted chemicals. Fe@TiO_2_ nanoparticles are dispersed into aqueous solution with concentration 0.05 g/mL. The dispersed Fe@TiO_2_ nanoparticle solution turned deep yellow with increasing Fe dopant concentration (see Additional file 1: Figure S1).

#### Preparation of the Fe@TiO_2_ Dispersed Nanoparticle Layers

Silicon wafers (1 cm × 1 cm) were washed with absolute ethanol, sonicated, and dried with an N_2_ stream followed by oxygen plasma treatment for 3 min. The dispersed Fe@TiO_2_ nanoparticle solution was then spin coated onto the silicon wafers at 2000 rpm. Subsequently, the spin-coated layers were annealed at 600 °C for 12 h at a heating rate of 5 °C/min in ambient atmosphere.

### Characterization

The X-ray diffractions (XRDs) of the Fe@TiO_2_ nanoparticles were obtained using a Rigaku D/Max-A diffraction meter by Ni-filtered Cu-K*α* radiation (40 kV, 300 mA). The scans were obtained by 4°/min with 0.01° step size. Raman spectra were obtained using a spectrometer (Horiba, LabRAM ARAMIS) with a 514.5-nm Ar ion CW laser. The morphologies of the samples were characterized by performing field-emission scanning electron microscopy (FE-SEM, FEI Inspect F50) at an acceleration voltage of 10 kV and field-emission transmission electron microscopy (FE-TEM, FEI Tecnai G^2^ F30 S-Twin) with energy dispersive X-ray spectroscopy (EDX) at an acceleration voltage of 300 kV. Scanning transmission X-ray microscopy (STXM) was performed at the 10A beamline at the Pohang Accelerator Laboratory (PAL). A Fresnel zone plate with an outermost zone width of 25 nm was used to focus the X-rays onto the Fe@TiO_2_ nanoparticles on the TEM grids. The transmitted intensity was measured with a scintillation-photomultiplier tube. Image stacks were acquired at 695–745, 450–480, and 520–570 eV using X-ray absorption spectroscopy (XAS) to extract the Fe *L*-edge, Ti *L*-edge, and O *K*-edge spectra, respectively. High-resolution photoemission spectroscopy (HRPES) experiments were performed at the PAL 8A1 beamline equipped with an electron analyzer (Physical Electronics, PHI-3057). The Fe 2*p*, Ti 2*p*, O 1*s*, and S 2*p* core level spectra were obtained using photon energies of 770, 510, 590, and 230 eV, respectively, to enhance the surface sensitivity. The binding energies of the core level spectra were determined with respect to the binding energy (*E*_B_ = 84.0 eV) of the clean Au 4*f* core level for the same photon energy.

### Photocatalytic Oxidation Reactions

2-Aminothiophenol (C_6_H_4_SHNH_2_, Sigma-Aldrich, 99 % purity) was purified by turbo pumping prior to dosing onto the Fe@TiO_2_ samples. A direct dozer controlled by a variable leak valve was used to dose the molecules with the same amount of oxygen molecules onto the Fe@TiO_2_ nanoparticles. UV-visible light (*λ* = 365 nm, 550 nm) exposure was maintained at 8 W through the vacuum chamber quartz window. Chamber pressure was maintained at 1 × 10^−6^ Torr during dosing, and the number of exposed molecules was defined by the dosing time in seconds: 1 L (Langmuir) corresponds to 1 s dosing at 1 × 10^−6^ Torr.

## Results and Discussion

**Fig. 1:**
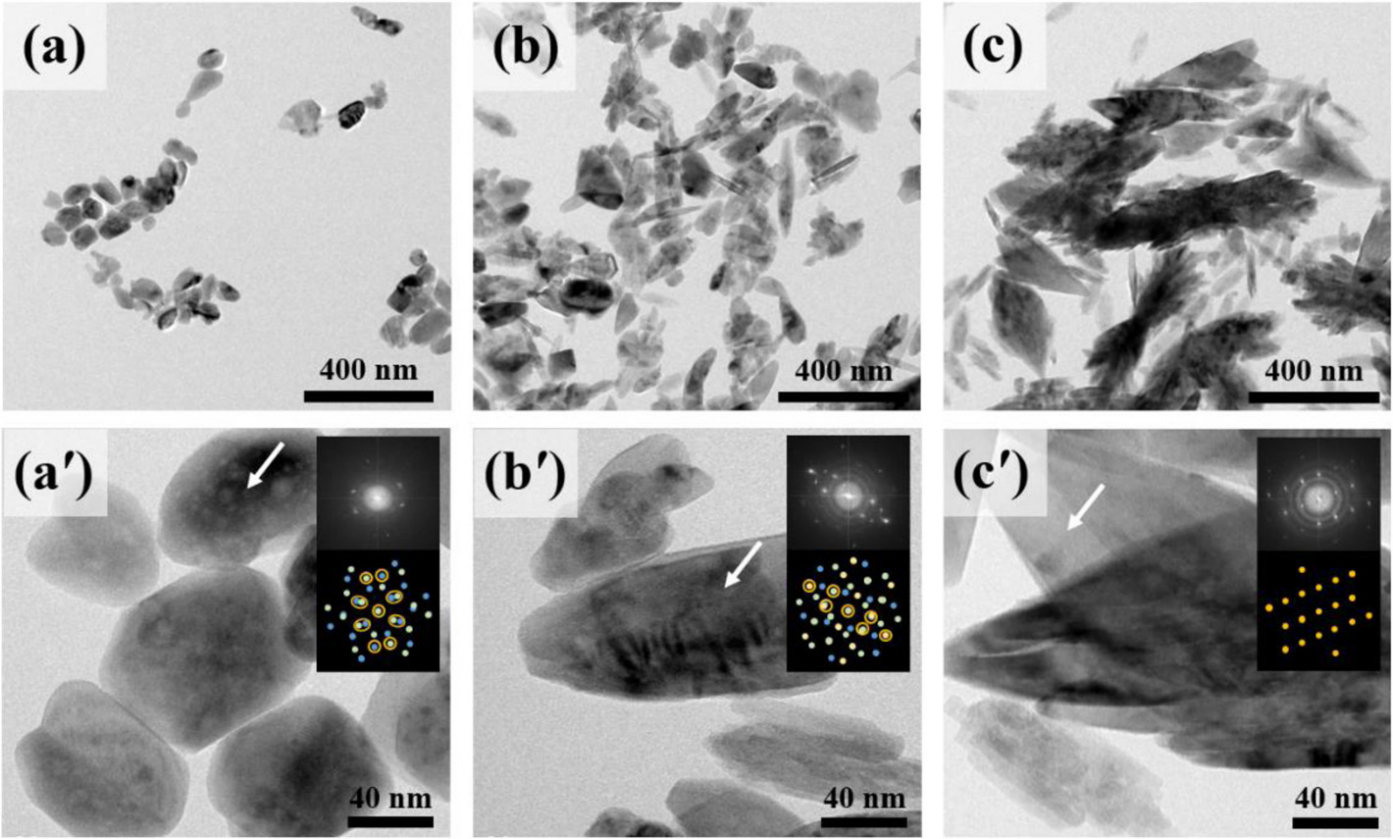
TEM images. **a** 1, **b** 3, and **c** 5 wt.%, and corresponding high-resolution images (*a′*), (*b′*), and (*c′*). *White arrows* indicate the location of inset diffraction patterns

We first acquired TEM (Fig. 1) and SEM (Additional file 1: Figure S2) images varying the weight percent of the Fe dopants. SEM images were obtained from the Fe@TiO_2_ on Si substrate, and TEM images were obtained from the powder Fe@TiO_2_ by using a similar method. According to the morphology of both images, they show the same structural feature. When 1 wt.% was doped inside TiO_2_, very fine (70 nm) and homogeneous circular Fe@TiO_2_ particles are evident. These particles are much smaller than bare TiO_2_ particles (~200 nm). It is expected that the Fe ions act as nucleation sites and crystallize to particles as a fine structure. When the Fe dopant concentration was increased, particles with fine needle-like structure and big arrow structure are evident.

**Fig. 2:**
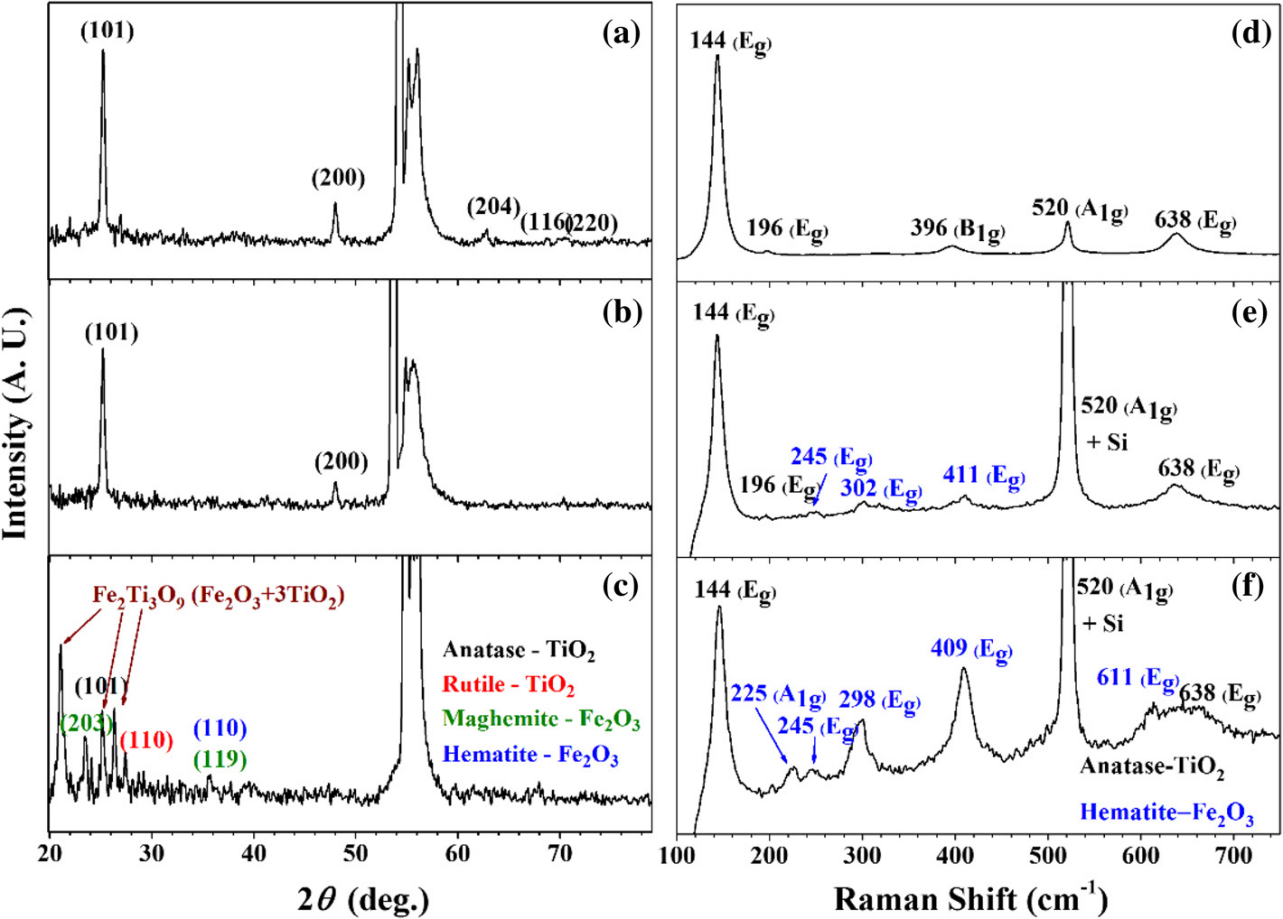
XRD and corresponding Raman spectra show that the crystal structures and chemical states vary with the amount of Fe dopant. **a**, **d** 1 wt.% Fe@TiO_2_. **b**, **e** 3 wt.% Fe@TiO_2_. **c**, **f** 5 wt.% Fe@TiO_2_

According to the fast Fourier transform (FFT) pattern in the upper inset of the high-resolution TEM images, the big arrow structures in each image correspond to the highly ordered TiO_2_ structure in which particle grains are overlaid with other crystalline directions and indicate different FFT patterns. The expected pattern overlaps are indicated in the lower inset of Fig. 1. These big arrow structures were regarded as pure or low concentration Fe@TiO_2_ particles. When the Fe concentration increases to 3 and 5 wt.%, small needle structures are evident on both SEM and TEM images. Such structures are obtained as Fe_2_O_3_ complex by exclusion of Fe ions from the arrows structures. According to Additional file 1: Figure S3, EDX spectra show the portion of Fe atom in TiO_2_ substrates. At 5 wt.%, large arrow structures contain almost no Fe atom which also support the exclusion of Fe atom from the big arrow structures. Thus, these small needles (120 nm × 40 nm) are composed by bundling of smaller units, such as Fe_2_O_3_ and TiO_2_ structures. Those structures are dominant in 5 wt.% Fe@TiO_2_. XRD and Raman analyses (Fig. 2) elucidate the structural features with variation of concentration.

**Fig. 3:**
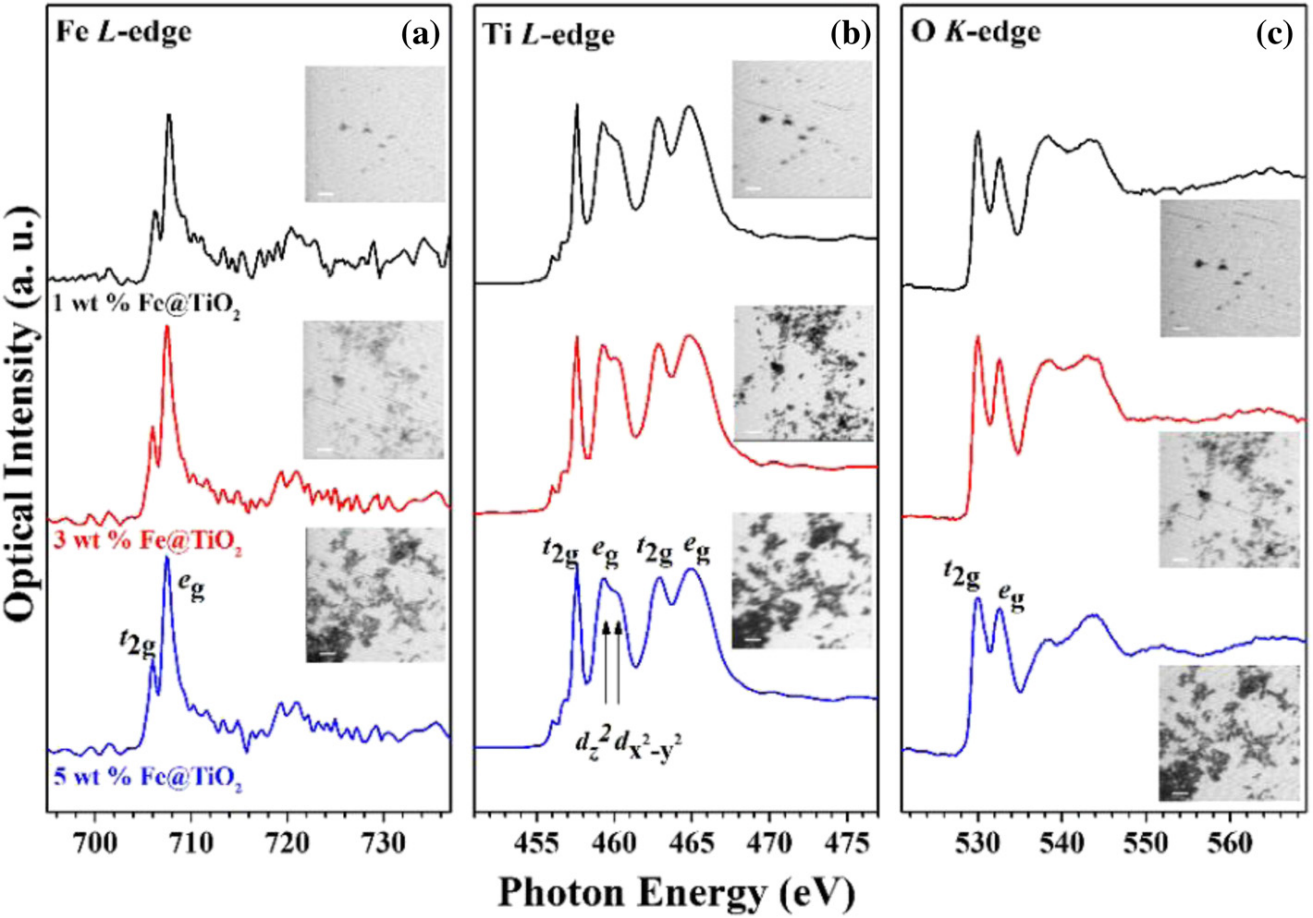
XAS spectra of **a** Fe *L*-edge, **b** Ti *L*-edge, and **c** O *K*-edge of 1 wt.% Fe@TiO_2_ (*black line*), 3 wt.% Fe@TiO2 (*red line*), and 5 wt.% Fe@TiO2 (*blue line*). *Insets* are STXM images of each spectrum

According to the XRD and Raman spectra shown in Fig. 2, anatase TiO_2_ structure is observed not only from 2*θ* = 25.27° and 48.05° with XRD (JCPDS#84-1286), which are associated with (101) and (200), but also from 144 (*E*_*g*_), 196 (*E*_*g*_), 392 (*B*_1*g*_), 520(*A*_1*g*_), and 638 (*E*_*g*_) on the Raman spectra [17]. Those clearly identify the structure of anatase TiO_2_ with no polymorph features. According to the XAS spectra from STXM (Fig. 3), the ratio between two *e*_*g*_ peaks of the Ti *L*-edge shows higher absorption of *d*_*z*_^2^ (459.1 eV) compared to *d*_*x*_^2^_− *y*_^2^ (460.0 eV), which is a typical XAS spectrum of the anatase TiO_2_ phase.

At 3 wt.% of Fe ions, the hematite-Fe_2_O_3_ structure appeared in the Raman spectra. When only a few layers of Fe_2_O_3_ were coated on the TiO_2_ surface, no XRD peak features were evident; however, coating with a small number of layers of Fe_2_O_3_ can cause lattice distortion. Additional file 1: Figure S4 shows the variation of the XRD peak position with the associated lattice constant of the (101) facet and the relative Raman intensity of *E*_*g*_ (410)/*E*_*g*_ (144) to elucidate the doping effects on structures. At 1 and 2 wt.% of Fe dopants, there are no Raman features with respect to Fe_2_O_3_, changing the lattice constant on XRD. This change occurs with the successful doping of Fe on the Ti substitutional sites. Such doping triggers the formation of oxygen vacancies to compensate the regional oxidation balance, which causes lattice contraction. At 3 wt.% of Fe dopants, the Raman spectrum of the hematite phase initially appears at 225, 245, 298, 409, and 611, which is associated with *A*_1*g*_ + 4*E*_*g*_ [18]. Furthermore, the lattice constant of the (101) facet expands too close to the original phase. This means that the precipitation can be attributed to the phase separation between Fe_2_O_3_ and TiO_2_. At a higher doping concentration (5 wt.%), very small features of the anatase phase are shown by XRD. On the other hand, new structural features are indicated at 21.08° and 26.35°. These new structural features are contributions from the formation of a TiO_2_ composite with Fe_2_O_3_. According to previous investigations, the composite of (Cr, Fe)_2_Ti_*n* − 2_O_2*n* − 1_ indicates a monoclinic system, and their intergrowth can be attributed to the *d*-spacing changes from 18.9° to 22° as 2*θ*. In particular, in the case of *n* = 5, *d*-spacing near 25° and 27° is also evident. Thus, the spectrum is characterized as Fe_2_Ti_3_O_9_ (Fe_2_O_3_ 3TiO_2_) [19–21].

The differences among the electronic structures of the samples were characterized by XAS measurements using STXM (Fig. 3). O *K*-edge XAS spectra has four peaks at 529.9, 532.3, 537.9, and 543.7 eV. Two peaks at 529.9 and 532.3 eV are attributed to the transition from O 1*s* orbital to the O 2*p* orbital hybridized with Ti 3*d t*_2*g*_ and *e*_*g*_ states, respectively. Two additional peaks at 537.9 and 543.7 eV are attributed to the delocalized state of the Ti 4*sp* and O 2*p* bands. At 3 to 5 wt.% dopant concentrations, the peak at 532.3 eV is reduced. This may be because of the presence of surface-oxygen-vacancy sites that cause reduction in Ti 3*d*–O 2*p* hybridization in *e*_*g*_ states and finally shows the reduction of intensity of 532.3 eV [22, 23]. This is also evident from the Ti *L*-edge spectra as well as the HRPES data.

Ti *L*_3,2_-edge XAS spectra show the traditional anatase TiO_2_ structure, which arises from the transitions of Ti 2*p* electrons to unoccupied 3*d* electronic states in a distorted octahedral crystal field. Two *t*_2*g*_, such as 457.4 and 462.7 eV, result from the transition of 2*p*_3/2_ and 2*p*_1/2_, respectively. Two *e*_*g*_ bands, i.e., 459–460 and 464.8 eV, can be attributed to the transition from the 2*p*_1/2_. When Fe ions are doped inside the TiO_2_, the intensity ratio of peaks *t*_2*g*_ (457.4 eV) to *e*_*g*_ (459~460 eV) is gradually lowered compared with that of 1 wt.% Fe@TiO_2_ nanoparticles, which indicates a weak crystal field or an increment of the number of under-coordinated Ti atoms [24, 25]. Furthermore, the ratio of peaks *d*_*z*_^2^ to *d*_*x*_^2^_− *y*_^2^ indicates the distortion of the octahedral crystal field, which is noticeable in the discrepancy between the rutile and anatase structures. These ratios also decrease as the percentage of Fe dopants increases. Therefore, the anatase structure transforms to less crystalline anatase by the formation of Fe_2_O_3_ 3TiO_2_. This transformation also gives rise to the surface defect structure, indicating that the small doublet at 456.0 and 456.6 eV is in the Ti^3+^ state [26].

*L*_3_ and *L*_2_-edges located at approximately 710 and 721 eV have been assigned as transitions from Fe 2*p* to an unoccupied 3*d* orbital. The distinct features at the *L*_3_-edge indicate typical *t*_2*g*_ (706.0 eV) and *e*_*g*_ (707.4 eV) splitting because of the presence of only the Fe^3+^ valence form. However, there is no noticeable oxygen state with respect to Fe_2_O_3_ [27–29].

**Fig. 4:**
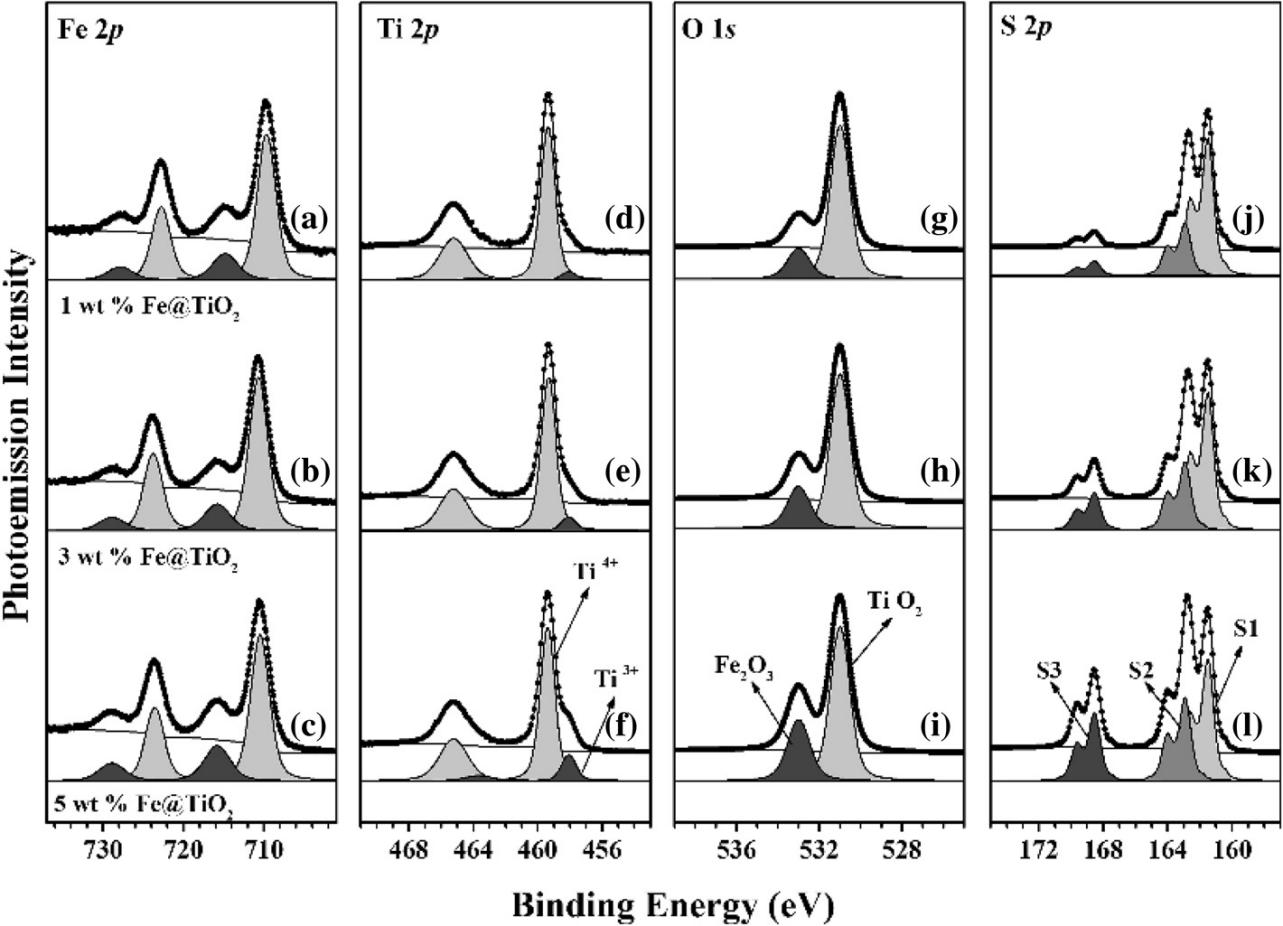
HRPES results for Fe 2*p*, Ti 2*p*, and O 1*s* core level spectra of Fe@TiO_2_ nanoparticles with various doping levels. **a**, **d**, **g** Core level spectra from 1 wt.% Fe@TiO_2_. **b**, **e**, **h** Those from 3 wt.% Fe@TiO_2_. **c**, **f**, **i** Those from 5 wt.% Fe@TiO_2_. HRPES results corresponding to the S 2*p* core level spectrum obtained after photocatalytic oxidation of 2-aminothiophenol on **j** 1, **k** 3, and **l** 5 wt.% Fe@TiO_2_ nanoparticles

The core level spectra (Fe 2*p*, Ti 2*p*, and O 1*s*) of the Fe@TiO_2_ nanoparticles were obtained with HRPES (Fig. 4) to determine the changes in electronic properties. According to the core level spectra of O 1*s*, the nanoparticles are composed of two different chemical environments assigned as TiO_2_ (530.9 eV) and Fe_2_O_3_ (533.0 eV), which correspond to the previous XRD, Raman, and XAS spectra. In Ti 2*p* core level spectra, there is a Ti^3+^ peak at 458.1 eV as well as the typical Ti^4+^ spectra of TiO_2_ at 459.4 eV. The ratio of Ti^3+^ peak to Ti^4+^ increases with the doping concentration, which is attributed to the oxygen-vacancy sites. When vacancies are generated, the oxidation state of the site is compensated by the nearest neighbor Ti through formation of Ti^3+^. This is also clearly evident from the pre-edge of the Ti *L*-edge and *e*_*g*_ intensity of the O *K*-edge in the XAS spectra. Fe shows Fe^3+^ characteristics at 710.7 eV, which is also comparable with the previous results.

**Fig. 5:**
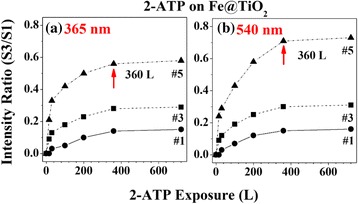
Plots of the ratios of S3 to S1, indicating photocatalytic activity by oxidation of 2-ATP as a function of molecular exposure under **a** 365-nm UV light and **b** 540-nm visible light

Next, we determined the photocatalytic activities of each Fe@TiO_2_ nanoparticle by the oxidation of 2-ATP molecules. The surface-sensitive S 2*p* core level spectra were recorded using HRPES after 360 L of 2-ATP exposure in the presence of the same amount of oxygen under 365-nm UV light illumination. There are three distinct 2*p*_3/2_ peaks at 161.5, 162.9, and 168.6 eV, which correspond to the C-SH unbounded state (denoted S1), bounded state (denoted S2), and sulfonic acid (SO_3_H) (denoted S3), respectively [30, 31]. Sulfonic acid was formed from the oxidation of thiol in 2-ATP. According to the S3 peaks of S 2*p*, sulfonic acid is increased as the concentration of Fe dopants in TiO_2_ increases. To confirm the effect of the energy of light and its photocatalytic performance, relative intensities were plotted as the ratio of S3 to S1 (Fig. 5).

Figure 5 shows the changes of relative intensity as a function of 2-ATP exposure under two different light sources. When UV (365 nm) is irradiated on the catalysts, photocatalytic activity increases by increase of Fe doping concentrates: 1 (0.15), 3 (0.29), and 5 wt.% (0.58). One significant observation is that photocatalytic oxidation is enhanced by increasing doping concentration under illumination in the visible range (540 nm). Otherwise, the ratio increased from 0.16 at 1 wt.% Fe to 0.73 at 5 wt.% of Fe, which shows significant increments compared to the intensity ratios under UV illumination. The phenomena arise from narrowing the TiO_2_ bandgap by doping and the heterojunction of Fe_2_O_3_ and TiO_2_ with high concentration.

**Fig. 6:**
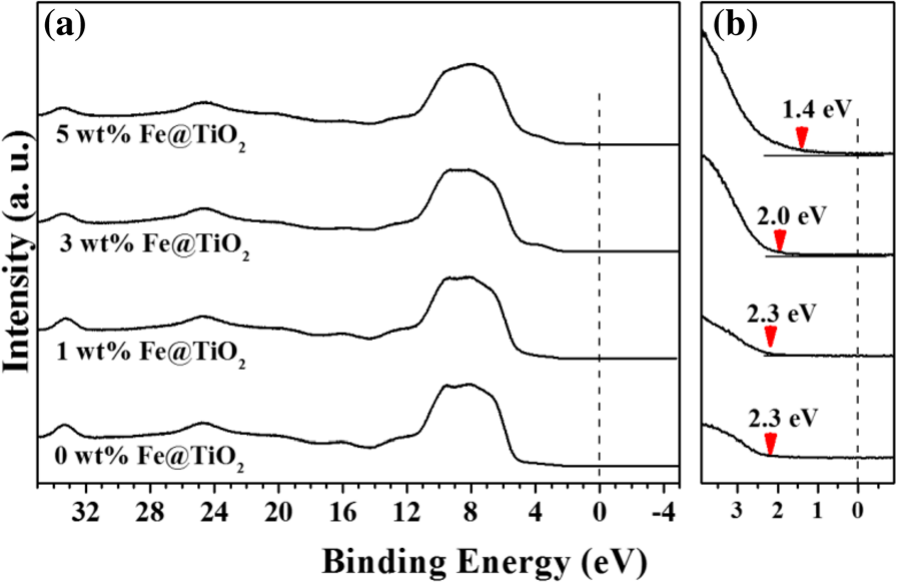
**a** Valence band spectra and **b** the Fermi-edge obtained at 0, 1, 3, and 5 wt.% of Fe-doped TiO_2_ nanoparticles (*marked*) from the photon energy of 80 eV

**Scheme 1:**
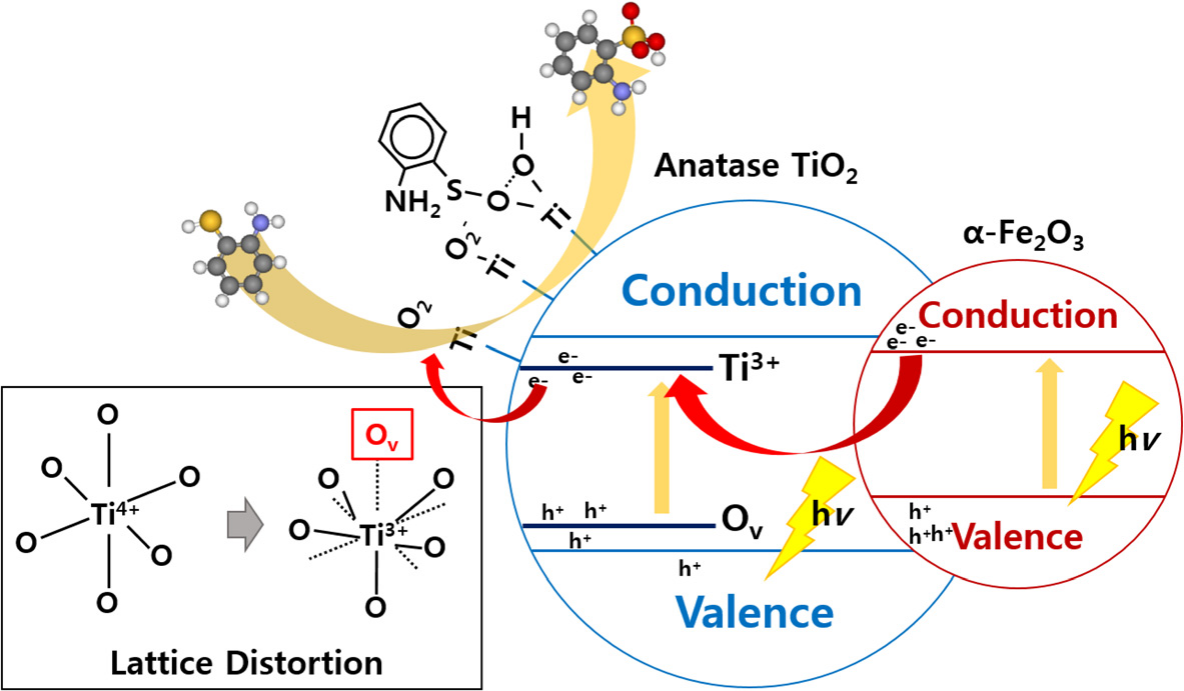
Schematic diagram for charge transfer with reaction mechanism

Figure 6 and Additional file 1: Figure S5 (subtracted spectra by valence band of bare-TiO_2_) show the valence band spectra and changes with variations of the Fermi-edge. As shown in the valence band spectra (Fig. 6b), density of states arises at the valence band maximum and is expanded from 2.3 to 2.0 eV, and then to 1.4 eV, which indicates that the density of states lowers the bandgap significantly. There are two significant reasons for the bandgap reduction when the doping concentration is increased to 5 wt.%. One is the Fe^3+^ doping inside the TiO_2_, which generates the surface states as well as the oxygen-vacancy sites observed by XAS spectra with Ti^3+^ states on the HRPES spectra [32]. Those Ti^3+^, oxygen-vacancy couples introduce the dopant level inside the bandgap below the conduction band minimum and above the valence band maximum, respectively. Calculated density of states of anatase TiO_2_ with one oxygen vacancy per 16 oxygen atoms shows the similar Fermi-edge at below 2 eV [33]. And controllable Ti^3+^ shows the significant reduction the bandgap [34, 35]. The second reason is the precipitation as hematite Fe_2_O_3_, where the particles have a lower bandgap (2.1 eV) compared to TiO_2_ (3.2 eV) and promote bandgap narrowing by heterojunction between Fe_2_O_3_ and TiO_2_, enhancing the absorption of visible light [8, 26, 36]. From the mechanistic point of view, generated electrons after light absorption are trapped to the Ti^3+^ state below the conduction band minimum which facilitates the charge separation by using heterojunction. Those trapped electrons are transferred to O_2_ species with formation of Ti-O_2_^−^ binding as peroxide. Peroxide has been indicated as electron scavenger O_2_ species in the traditional photocatalytic oxidation on TiO_2_ [37, 38]. S 2*p* spectra of HRPES (Fig. 4) show the relatively large amount of bound state (S2) when the doping level is increased. This is caused by the dissociative adsorption of –SH group at the oxygen atom of TiO_2_ which triggers the decomposition of peroxide [39, 40]. Consequently, the amount of trapped electrons at the Ti^3+^ state captures and stabilizes the O_2_ then 2-ATP decomposes –SH to –S-O-Ti (bound state, S2), –OH on the peroxide. (Scheme 1) –SH group of 2-ATP can be activated by the transfer of hole from the valence band. The energy relaxation of –SH group is around 2 eV compared to bare TiO_2_ which makes electron deficient –SH group to facilitate the oxidation reaction with electron rich peroxide [41]. Surface Fe^3+^ sites play a role of stabilization of –OH by hydrogen bonding during reaction process [42, 43]. From those reasons, doping concentration at 3 and 5 wt.% shows the narrowed bandgap that absorbs the photons more efficiently under 540 nm visible light, thus resulting in the significant enhancement of photo-oxidation activity [44, 45].

## Conclusions

In summary, Fe@TiO_2_ nanoparticles were synthesized by sol-gel using a hydrothermal method. Their photocatalytic activity was characterized using surface spectroscopic measurements. According to the XRD, Raman, XAS, and HRPES spectra, Fe ions are successfully doped inside the anatase TiO_2_ substrate, and the crystal structure is changed to a distorted anatase-like structure that also has a hematite-Fe_2_O_3_ character as a Fe_2_O_3_ 3TiO_2_ complex. These structural features form the lamellar structure in TEM images, which is attributed to the heterojunction between hematite Fe_2_O_3_ and anatase TiO_2_. Furthermore, the introduction of Fe^3+^ ions generates discrepancies in the oxidation state, which is compensated by the formation of oxygen vacancies and transforms to the surface [46]. These structural changes induce additional states at the edge of the conduction band and valence band, finally narrowing the bandgap. Moreover, the Ti^3+^ plays a role of electron trap to deliver the electrons to O_2_ species for oxidation reaction. As a result, photocatalytic oxidation activities of 2-ATP are significantly increased for both UV and visible photon energies.

## Additional file

Additional file 1: **Supplementary material.** Digital images and SEM images of Fe@TiO2 nanoparticles, XRD, and Raman intensity plot. **Figure S1.** Digital images of the Fe@TiO_2_ dispersed solution with several of Fe dopant concentration. **Figure S2.** SEM images as the morphologies varying the doping level of Fe: (a) 1 wt %, (b) 3 wt %, (c) 5 wt %; and their high-resolution images (a′), (b′), and (c′), respectively. **Figure S3.** EDX spectra with Fe peak (marked by black arrows) of big particles: (a) 1 wt% Fe@TiO_2_, (b) 3 wt% Fe@TiO_2_, and (c) 5 wt% Fe@TiO_2_. **Figure S4.** (a) XRD peak position and correspond lattice constant of (101) plane of anatase TiO_2_ structure and (b) Raman intensity ratio of I_410_ (α-Fe_2_O_3_ E_g_) to I_144_ (anatase TiO_2_ E_g_). **Figure S5.** Spectral subtraction of valence band spectra by bare TiO_2_ peak: (a) 1 wt% Fe@TiO_2_, (b) 3 wt% Fe@TiO_2_, and (c) 5 wt% Fe@TiO_2_.
