# Supplementary material for: Enhancement of Photo-Oxidation Activities Depending on Structural Distortion of Fe-Doped TiO2 Nanoparticles
Source: Nanoscale Res Lett. 2016 Jan 29;11:41. doi: 10.1186/s11671-016-1263-6 (PMC4731377; doi:10.1186/s11671-016-1263-6)
Supplement: Supplementary file 1 — Supplementary material. Digital images and SEM images of Fe@TiO2 nanoparticles, XRD, and Raman intensity plot. Figure S1. Digital images of the Fe@TiO2 dispersed solution with several of Fe dopant concentration. Figure S2. SEM images as the morphologies varying the doping level of Fe: (a) 1 wt %, (b) 3 wt %, (c) 5 wt %; and their high-resolution images (a′), (b′), and (c′), respectively. Figure S3. EDX spectra with Fe peak (marked by black arrows) of big particles: (a) 1 wt% Fe@TiO2, (b) 3 wt% Fe@TiO2, and (c) 5 wt% Fe@TiO2. Figure S4. (a) XRD peak position and correspond lattice constant of (101) plane of anatase TiO2 structure and (b) Raman intensity ratio of I410 (α-Fe2O3 Eg) to I144 (anatase TiO2 Eg). Figure S5. Spectral subtraction of valence band spectra by bare TiO2 peak: (a) 1 wt% Fe@TiO2, (b) 3 wt% Fe@TiO2, and (c) 5 wt% Fe@TiO2. [file 11671_2016_1263_MOESM1_ESM.docx]

**Supplementary Material**

Enhancement of Photo-oxidation Activities Depending on Structural Distortion of Fe-Doped TiO_2_ Nano-Particles

*Yeonwoo Kim,^1^ Sena Yang,^1^ Eun Hee Jeon,^1^ Jaeyoon Baik,^2^ Namdong Kim,^2^ Hyun Sung Kim,^3^* Hangil Lee ^4^**

Yeonwoo Kim^1^ Email : younwooj@kaist.ac.kr

Sena Yang^1^ Email : senacap@kaist.ac.kr

Eun Hee Jeon^1^ Email : ehj976@kaist.ac.kr

Jaeyoon Baik^2^ Email : cla100@postech.ac.kr

Namdong Kim^2^ Email : east@postech.ac.kr

Hyun Sung Kim^3^* Email : kimhs75@pknu.ac.kr

Hangil Lee ^4^* Email : easyscan@sookmyung.ac.kr

*Corresponding author

^1^ Molecular-Level Interfaces Research Center, Department of Chemistry, KAIST, Daejeon 34141, Republic of Korea

^2^ Beamline Research Division, Pohang Accelerator Laboratory (PAL), Pohang 37673, Kyungbuk, Republic of Korea

^3^ **Department of Chemistry, Pukyoung National University, Busan 48513,** Republic of Korea

^4^ Department of Chemistry, Sookmyung Women's University, Seoul 04310, Republic of Korea


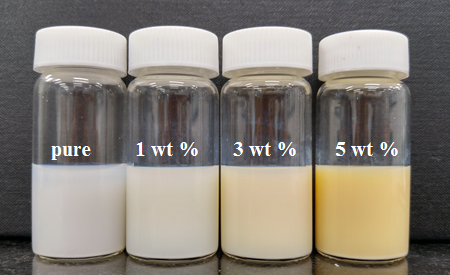


**Figure S1.** Digital images of the Fe@TiO_2_ dispersed solution with several of Fe dopant concentration.


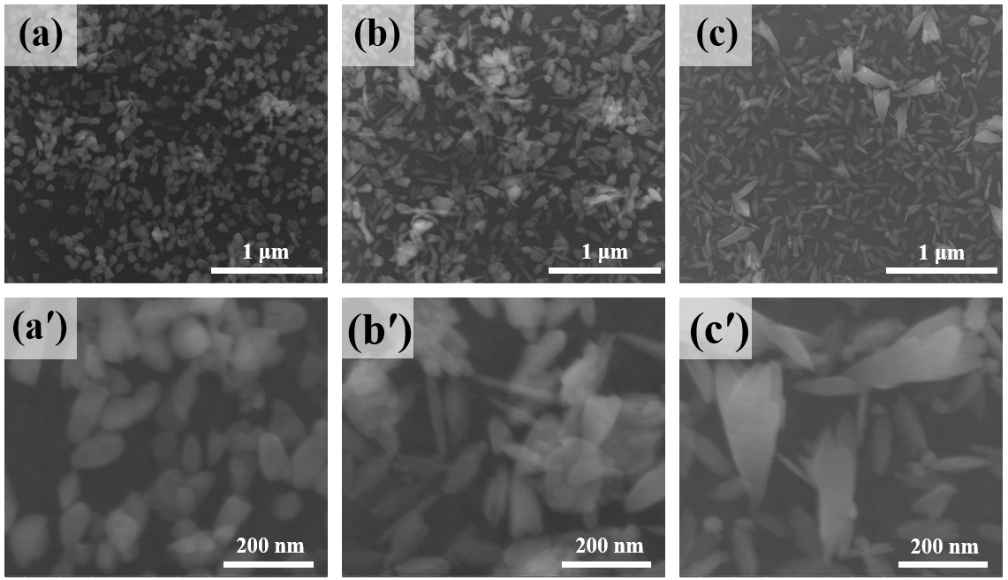


**Figure S2.** SEM images as the morphologies varying the doping level of Fe: (a) 1 wt %, (b) 3 wt %, (c) 5 wt %; and their high resolution images (a′), (b′), and (c′), respectively.


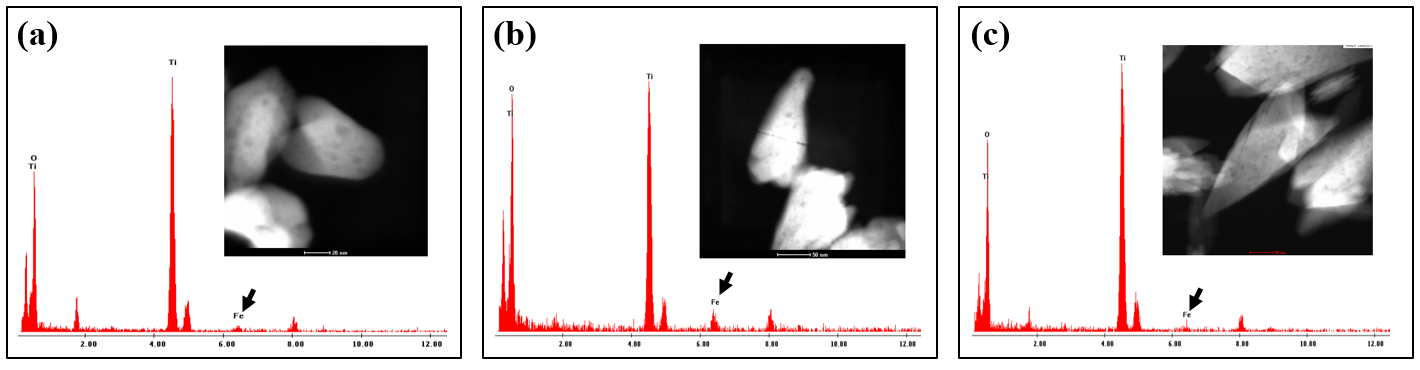


**Figure S3.** EDX spectra with Fe peak (marked by black arrows) of big particles: (a) 1 wt% Fe@TiO_2_, (b) 3 wt% Fe@TiO_2_, and (c) 5 wt% Fe@TiO_2_.


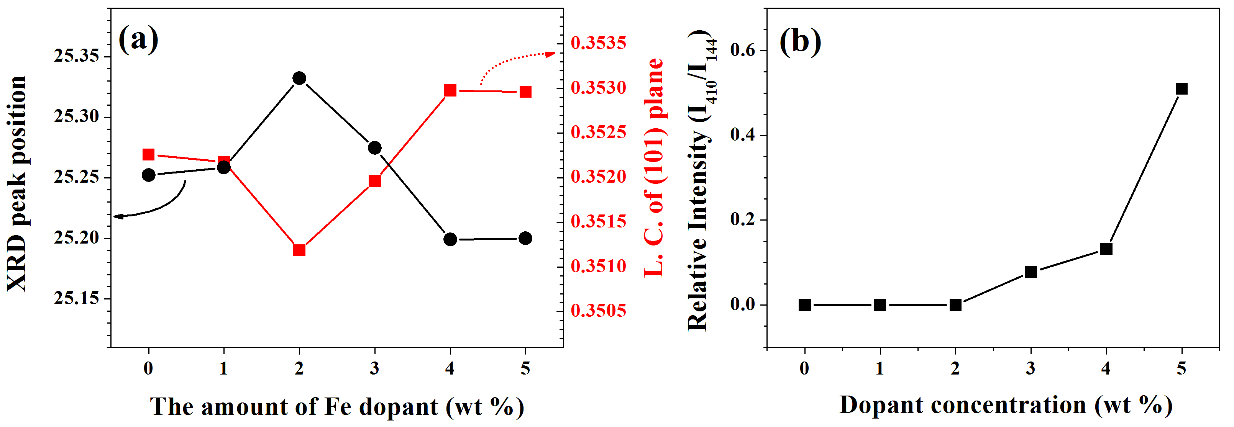


**Figure S4.** (a) XRD peak position and correspond lattice constant of (101) plane of anatase TiO_2_ structure and (b) Raman intensity ratio of I_410_ (α-Fe_2_O_3_ E_g_) to I_144_ (anatase TiO_2_ E_g_).


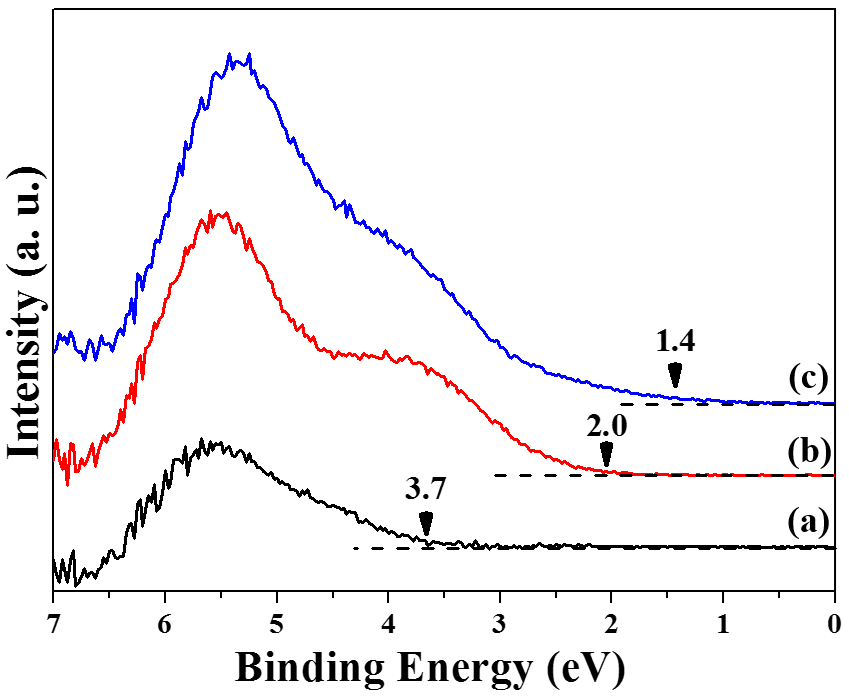


**Figure S5**. Spectral subtraction of valence band spectra by bare TiO_2_ peak: (a) 1 wt% Fe@TiO_2_, (b) 3 wt% Fe@TiO_2_, and (c) 5 wt% Fe@TiO_2_.
